# Caveolin-1 controls mitochondrial function through regulation of *m*-AAA mitochondrial protease

**DOI:** 10.18632/aging.101051

**Published:** 2016-10-04

**Authors:** Daniela Volonte, Zhongmin Liu, Sruti Shiva, Ferruccio Galbiati

**Affiliations:** ^1^ Department of Pharmacology and Chemical Biology, University of Pittsburgh School of Medicine, Pittsburgh, PA 15261, USA; ^2^ Vascular Medicine Institute and Center for Metabolism and Mitochondrial Medicine, University of Pittsburgh School of Medicine, Pittsburgh, PA 15261, USA

**Keywords:** caveolin-1, caveolae, oxidative stress, mitochondria, glycolysis

## Abstract

Mitochondrial proteases ensure mitochondrial integrity and function after oxidative stress by providing mitochondrial protein quality control. However, the molecular mechanisms that regulate this basic biological function in eukaryotic cells remain largely unknown. Caveolin-1 is a scaffolding protein involved in signal transduction. We find that AFG3L2, a *m*-AAA type of mitochondrial protease, is a novel caveolin-1-interacting protein *in vitro*. We show that oxidative stress promotes the translocation of both caveolin-1 and AFG3L2 to mitochondria, enhances the interaction of caveolin-1 with AFG3L2 in mitochondria and stimulates mitochondrial protease activity in wild-type fibroblasts. Localization of AFG3L2 to mitochondria after oxidative stress is inhibited in fibroblasts lacking caveolin-1, which results in impaired mitochondrial protein quality control, an oxidative phosphorylation to aerobic glycolysis switch and reduced ATP production. Mechanistically, we demonstrate that a lack of caveolin-1 does not alter either mitochondrial number or morphology but leads to the cytoplasmic and proteasome-dependent degradation of complexes I, III, IV and V upon oxidant stimulation. Restoration of mitochondrial respiratory chain complexes in caveolin-1 null fibroblasts reverts the enhanced glycolysis observed in these cells. Expression of a mutant form of AFG3L2, which has reduced affinity for caveolin-1, fails to localize to mitochondria and promotes degradation of complex IV after oxidative stress. Thus, caveolin-1 maintains mitochondrial integrity and function when cells are challenged with free radicals by promoting the mitochondrial localization of *m*-AAA protease and its quality control functions.

## INTRODUCTION

Mitochondria regulate the bioenergetic capacity of most eukaryotic cells. A major biological function of mitochondria is the production of adenosine tri-phosphate (ATP) through cellular respiration. This is accomplished by transferring electrons through mitochondrial respiratory chain complexes, which is coupled with the transferring of protons out of the matrix space. This creates a gradient that drives protons back through the membrane through ATP synthase, which allows the synthesis of ATP from ADP [1]. Since mitochondria are sensitive targets of reactive oxygen species (ROS), eukaryotic cells have developed several mitochondrial protein quality control systems for the maintenance of protein homeostasis and the prevention of oxidant-induced mitochondrial damages [2]. These include mitochondrial proteases, which degrade, process and mediate the assembly and disassembly of mitochondrial macromolecular structures involved in a variety of mitochondrial activities, including oxidative phosphorylation [3].

The matrix-oriented AAA (*m*-AAA) protease belongs to the ATPase associated with diverse cellular activities + (AAA+) family of mitochondrial proteases. It exists as a heterologous complex composed of AFG3L2 and paraplegin [4, 5]. *m*-AAA regulates the maintenance of aerobic respiration by providing mitochondrial protein quality control. Moreover, *m*-AAA has been proposed as a regulator of mitochondrial protein synthesis and network integrity through its ability to proteolytically process key substrates [6, 7]. Yeast deficient of *m*-AAA have dramatic respiratory defects and reduced ATP synthesis due to disrupted assembly of respiratory chain complexes [8–11]. Similarly, knockdown of AFG3L2 in human fibroblasts results in reduced respiratory complex levels [12]. In addition, human cells lacking *m*-AAA are more sensitive to ROS-induced mitochondrial damage and display reduced ATP synthesis [6]. Thus, mitochondrial *m*-AAA protease activity is necessary to eliminate ROS-damaged mitochondrial proteins in order to maintain mitochondrial functions such as aerobic respiration. Importantly, in the absence of the beneficial mitochondrial *m*-AAA activity, a seemingly paradoxical proteolytic degradation of mitochondrial proteins, triggered by oxidative stress, occurs in cells leading to the accumulation of nonfunctional mitochondria. However, the molecular mechanisms through which *m*-AAA protease is regulated under conditions of oxidative stress remain poorly understood.

Caveolin-1 is the structural protein component of caveolae, invaginations of the plasma membrane [13, 14]. Caveolin-1 acts as a scaffolding protein that concentrates and functionally regulates signaling molecules [15–24]. Our laboratory was the first to show that sublethal doses of oxidative stress upregulate caveolin-1 protein expression [20, 23] and increase the number of caveolae in fibroblasts [22] in a time-dependent manner. We demonstrated that oxidants stimulate caveolin-1 gene transcription through p38 mitogen-activated protein kinase/Sp1-mediated activation of two GC-rich promoter elements [20, 23]. Quercetin and vitamin E, two antioxidant agents, prevented the upregulation of caveolin-1 induced by oxidative stress [23]. Consistent with these data, upregulation of caveolin-1 by oxidative stress was reported in cartilage [25] and endothelial cells [26]. Oxidative stress is a known activator of NF-κB. Interestingly, the intronic region of caveolin-1 contains NF-κB consensus sites and data show that LPS activation of endothelial cells increases caveolin-1 protein expression in an NF-κB-dependent manner [27]. Thus, it is possible that oxidants also promote transcription of the caveolin-1 gene promoter in an NF-κB-mediated manner. Upregulation of caveolin-1 by oxidants leads to the regulation of a number of signaling pathways. For example, we have shown that caveolin-1 modulates Mdm2 [19], ATM [24], PP2A-C [24], Nrf2 [28] and Sirt1 [29] following oxidative stress in fibroblasts. Others have reported that oxidative stress regulates signaling mediated by nitric oxide [30], HIF-1ɑ [31], STAT3 [32] and heme oxygenase 1 (HO-1) [33] through caveolin-1. Together, these data indicate that caveolin-1 is an oxidant-induced protein and that caveolin-1-mediated signaling is activated in response to oxidative stress.

Interestingly, transmission electron microscopy showed that caveolae establish contacts with mitochondria [34]. In addition, caveolin-1 immunogold electron micro-scopy demonstrated that caveolin-1 partially localizes to mitochondria [35]. In support of these findings, mitochondrial proteins were identified in purified caveolae by proteomic analysis [36] and caveolin was found in mitochondria by immuno-fluorescence and immunoblotting analysis [34, 35, 37]. Thus, caveolin-1 may have an underappreciated role as a regulator of mitochondrial functions. Since oxidative stress upregulates caveolin-1 protein expression and given the notion that mitochondria possess quality control systems to avoid oxidant-induced damage, we investigated the hypothesis that caveolin-1-mediated signaling is activated under conditions of oxidative stress to maintain mitochondrial function. We found that caveolin-1 is a novel endogenous binding partner of AFG3L2. We show that caveolin-1 promotes the localization of AFG3L2 to mitochondria after oxidative stress, where AFG3L2 maintains mitochondrial function by providing mitochondrial protein quality control. In the absence of caveolin-1 expression, AFG3L2 fails to move to mitochondria upon oxidant stimulation. As a consequence, a lack of mitochondrial protein quality control leads to degradation of mitochondrial respiratory chain complexes and a switch from oxidative phosphorylation to glycolysis. Together, our data provide novel mechanistic insights into how cells prevent bioenergetic dysfunctions when exposed to oxidative stress.

## RESULTS

### AFG3L2 is a caveolin-1-binding protein

**Figure 1:**
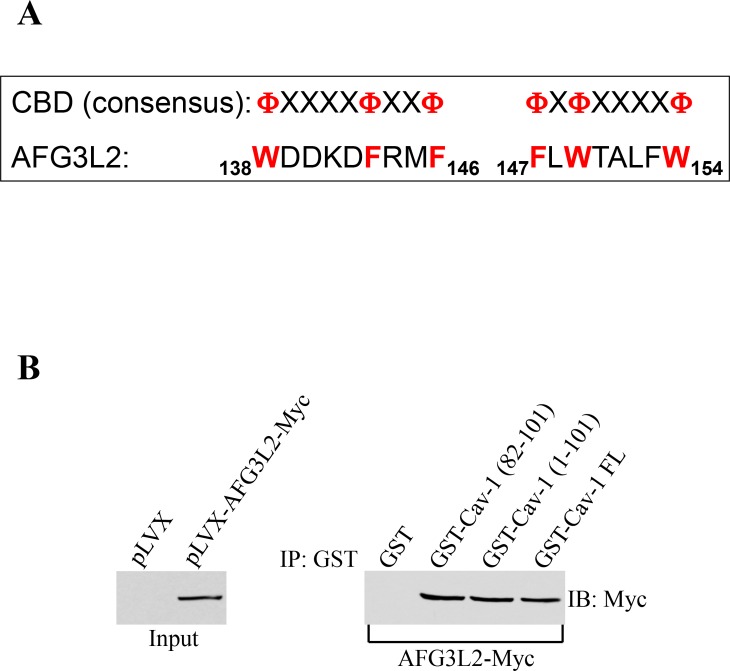
AFG3L2 directly interacts with caveolin-1 *in vitro* (**A**) Schematic diagram showing the consensus caveolin-binding-domain (CBD) and the CBD of human AFG3L2 (amino acids 138-146 and 147-154). F represents an aromatic amino acid and X represents any amino acid. (**B**) Caveolin-1-GST fusion proteins [GST-Cav-1(82-101), GST-Cav-1(1-101) and GST-Cav-1-FL] were used in pull down assays with cell lysates from NIH 3T3 cells transiently transfected with AFG3L2-myc. Pull-down assays with GST alone was used as internal control.

The scaffolding domain of caveolin-1 (CSD), which is represented by residues 82-101, mediates direct protein-protein interactions between caveolin-1 and a variety of signaling molecules carrying the caveolin binding domain (CBD: FXFXXXXF, FXXXXFXXF, or FXFXXXXFXXF where F represents an aromatic amino acid and X represents any amino acid) [38–40]. Analysis of the AFG3L2 protein sequence indicates that AFG3L2 has two putative caveolin-binding-domains between amino acids 138 and 146 and between amino acids 147 and 154 (Figure 1A). Thus, to investigate whether caveolin-1 is a binding partner of AFG3L2, we performed pull-down assays using a series of caveolin-1 deletion mutants fused to GST. Figures 1B shows that AFG3L2 was a caveolin-1-binding protein and that the scaffolding domain of caveolin-1 was sufficient for binding to AFG3L2.

### Caveolin-1 promotes the accumulation of AFG3L2 to mitochondria after oxidative stress

We have previously shown that sub-cytotoxic levels of oxidants up-regulate caveolin-1 protein expression [20, 22, 23] and activate caveolin-1-mediated signaling [19, 21, 24, 28, 29]. We show in Figure 1 that caveolin-1 interacted with AFG3L2 *in vitro*. Given the notion that mitochondria possess quality control systems to avoid oxidant-induced damage, we started to investigate the hypothesis that caveolin-1-mediated signaling is activated under conditions of oxidative stress to maintain mitochondrial function by asking whether sublethal levels of oxidants promote the interaction of caveolin-1 with AFG3L2 in cells.

**Figure 2:**
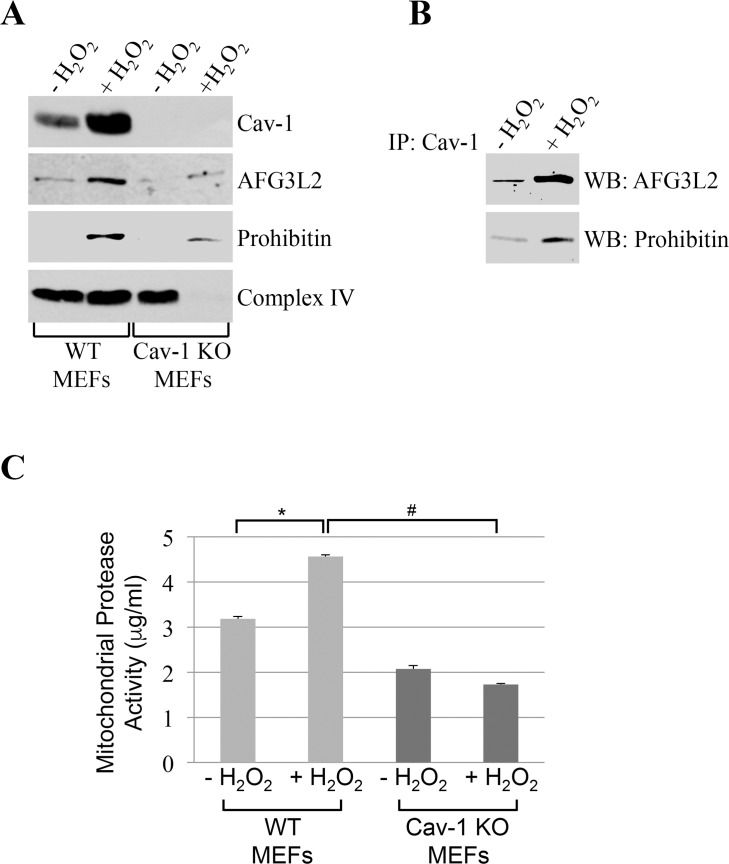
AFG3L2 interacts with caveolin-1 after oxidative stress *in vivo* Wild type and caveolin-1 null mouse embryonic fibroblasts (MEFs) were treated with sublethal doses of hydrogen peroxide (150 μM) for 2 hours. Cells were then recovered in complete medium for 7 days. We have chosen these conditions because we have previously shown that they upregulate caveolin-1 expression [20, 22, 23] and activate caveolin-1-mediated signaling [19, 21, 24, 28, 29]. Untreated cells (−H_2_O_2_) were used as control. (**A**) Mitochondrial fractions were isolated and the expression levels of caveolin-1, prohibitin-1 and AFG3L2 were measured by immunoblotting analysis. (**B**) Mitochondrial fractions were isolated from untreated and hydrogen peroxide-treated wild type MEFs and immunoprecipitated using an antibody probe specific for caveolin-1 (Cav-1); immunoprecipitates were then subjected to immunoblotting analysis with anti-AFG3L2 and prohibitin-1 IgGs. (**C**) Mitochondria were isolated and mitochondrial protease activity was quantified using the Protease Fluorescent Detection Kit from Sigma-Aldrich (St. Louis, MO) (PF0100). Values in (**C**) represent mean ± SEM; *^,#^*P*<0.001.

We found that caveolin-1 and AFG3L2 were only marginally found in mitochondrial fractions under resting conditions in mouse embryonic fibroblasts (MEFs) (Figure 2A). However, after sublethal oxidative stress, both caveolin-1 and AFG3L2 translocated to mitochondria in MEFs (Figure 2A). Importantly, co-immunoprecipitation studies show that caveolin-1 interacted with AFG3L2 in mitochondria and that their interaction was enhanced by oxidative stress (Figure 2B). Interestingly, the oxidant-induced localization of AFG3L2 to mitochondria was dramatically inhibited in MEFs lacking caveolin-1 expression (Figure 2A). We conclude that caveolin-1 expression is necessary for the accumulation of AFG3L2 to mitochondria when cells are challenged by oxidants.

### Mitochondrial protease activity is reduced in cells lacking caveolin-1

What is the functional consequence of reduced accumulation of AFG3L2 in mitochondria, after oxidative stress, in cells lacking caveolin-1? Since AFG3L2 is a major mitochondrial protease, we assessed mitochondrial protease activity in cells either expressing or lacking caveolin-1 before and after oxidative stress. We found that oxidative stress enhanced mitochondrial protease activity in wild type MEFs (Figure 2C). In contrast, oxidant stimulation failed to increase mitochondrial protease activity in cells lacking caveolin-1 (Figure 2C), in which AFG3L2 did not accumulate in mitochondria (Figure 2A).

### Oxidative stress induces bioenergetic dysfunctions in cells lacking caveolin-1

**Figure 3:**
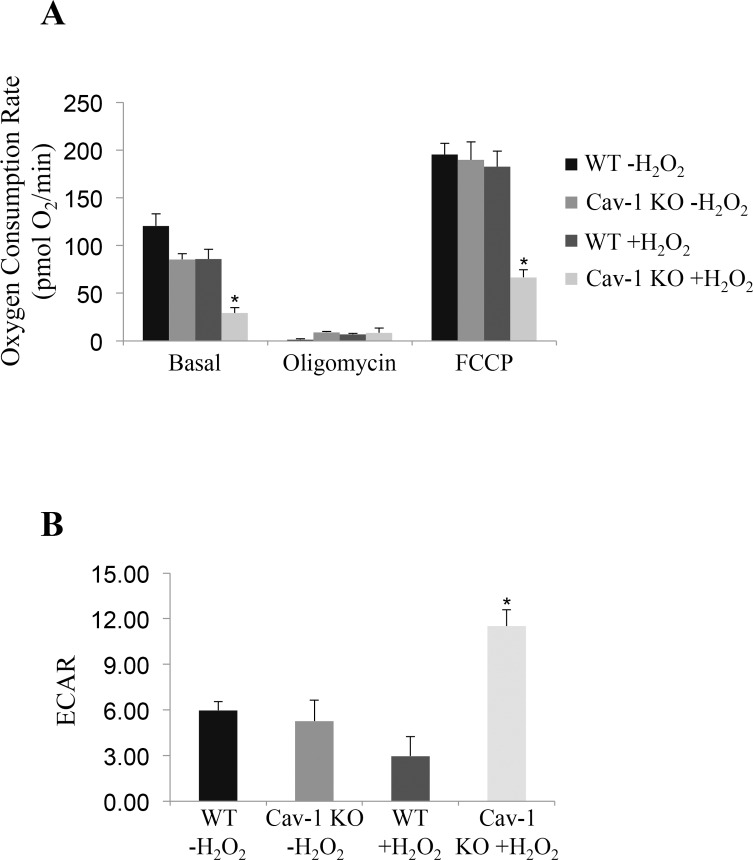
A lack of caveolin-1 promotes bioenergetic defects in ROS-treated fibroblasts Wild type and caveolin-1 null mouse embryonic fibroblasts (MEFs) were treated with sublethal doses of hydrogen peroxide (150 μM) for 2 hours. Cells were then recovered in complete medium for 7 days. Untreated cells (−H_2_O_2_) were used as control. Bioenergetic profile was determined using the Seahorse Metabolic Analyzer, which simultaneously measures oxygen consumption rate (OCR) (**A**) and extracellular acidification rate (ECAR) (**B**). Viability was assessed by crystal violet staining. OCR and ECAR were normalized to cell number. Values in (**A**) and (**B**) represent mean ± SEM; *P<0.001.

**Figure 4:**
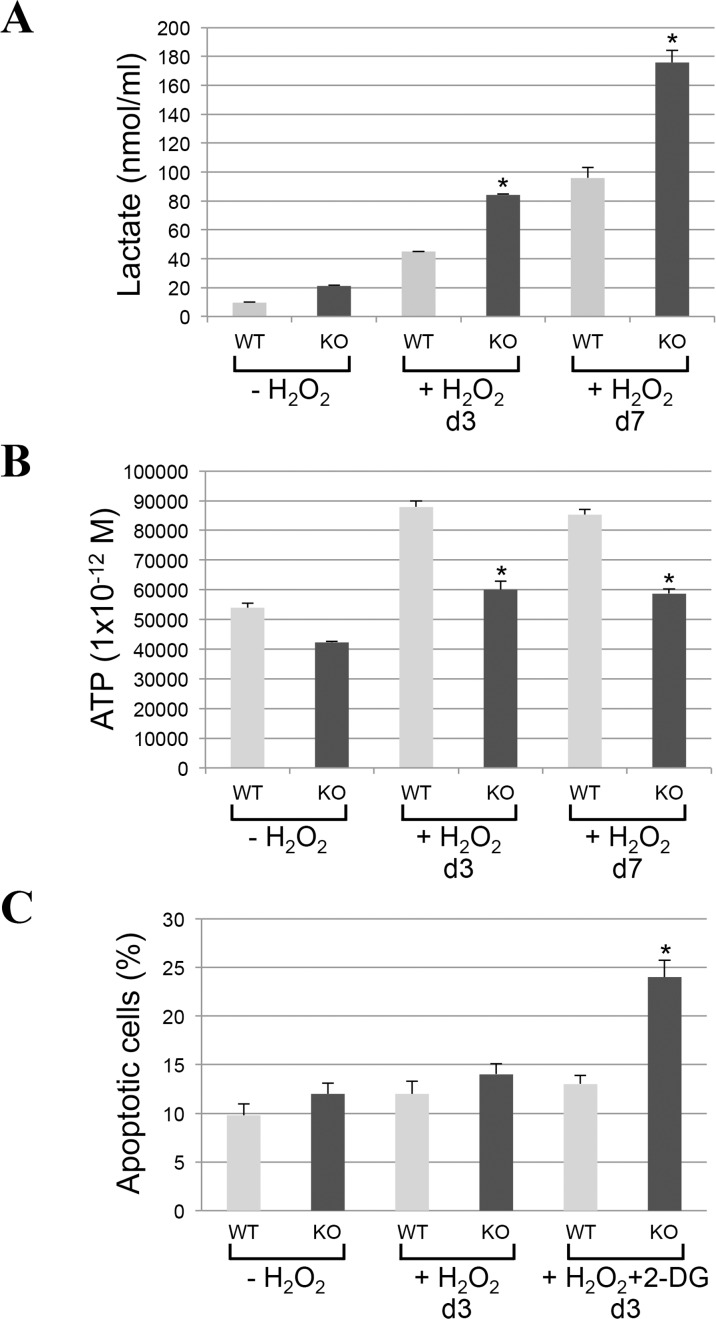
Lactate production is increased and ATP synthesis is inhibited in caveolin-1-lacking fibroblasts following oxidative stress (**A-B**) Wild type and caveolin-1 null mouse embryonic fibroblasts (MEFs) were treated with sublethal doses of hydrogen peroxide (150 μM) for 2 hours. Cells were then recovered in complete medium for different periods of time (3 days and 7 days). Untreated cells (−H_2_O_2_) were used as control. (**A**) Lactate production was quantified using the Lactate Assay Kit from Sigma-Aldrich (MAK064). (**B**) ATP production was quantified using the Adenosine 5′-triphosphate (ATP) Bioluminescent Assay Kit from Sigma-Aldrich (FL-AA). Values were normalized to cell number. (**C**) Wild type and caveolin-1 null mouse embryonic fibroblasts (MEFs) were treated with sublethal doses of hydrogen peroxide (150 μM) for 2 hours in the presence or absence of 2-deoxy-D-glucose (2-DG; 5mM). Cells were recovered in complete medium for 3 days in the presence or absence of 2-DG. Untreated cells (−H_2_O_2_) were used as control. Cells were stained with DAPI and the number of cells showing nuclear condensation was quantified. Values in (**A-C**) represent mean ± SEM; *P<0.001.

Mitochondrial proteases regulate mitochondrial protein quality control. Since impaired mitochondrial protease activity has been associated with mitochondrial respiratory defects and we found that AFG3L2 does not accumulate in mitochondria in caveolin-1 null cells after oxidative stress, we asked whether a lack of caveolin-1 leads to bioenergetic dysfunctions. Bioenergetic profile of wild type and caveolin-1 null MEFs, under resting conditions and after sublethal oxidative stress, was determined using the Seahorse Metabolic Analyzer, which simultaneously measures oxygen consumption rate (OCR) and extracellular acidification rate (ECAR). OCR is an indicator of mitochondrial respiration while ECAR is predominantly the result of glycolysis. We found that both the basal and maximal oxygen consumption rates were dramatically inhibited only in caveolin-1 null MEFs after oxidative stress (Figure 3A). In addition, our data show that ECAR was significantly elevated only in caveolin-1 null cells following oxidant stimulation (Figure 3B). Consistent with these data, we show that lactate production, a direct indication of glycolysis, is significantly higher in caveolin-1 null MEFs, as compared to wild type MEFs, after oxidative stress (Figure 4A). Since the number of ATP molecules produced through glycolysis is lower than that produced through oxidative phosphorylation, one would expect reduced ATP production in cells lacking caveolin-1. Consistent with this prediction, we show in Figure 4B that production of ATP is inhibited in caveolin-1 null MEFs, as compared to wild type cells. Together, these data indicate that a lack of caveolin-1 promotes an oxidative phosphorylation to glycolysis metabolic switch under conditions of oxidative stress. Finally, since a switch from oxidative phosphorylation to glycolysis makes cells more sensitive to glucose deprivation, we assessed the dependence from glucose of wild type and caveolin-1 null MEFs after oxidative stress. To this end, cells were treated with 2-deoxy-D-glucose, a glucose molecule that does not undergo glycolysis and mimics glucose deprivation. We show in Figure 4C that the percentage of apoptotic cells was significantly higher when caveolin-1 null MEFs where subjected to oxidant stimulation in the presence of 2-deoxy-D-glucose, as compared to wild type MEFs.

### Mitochondrial respiratory chain complexes are degraded in caveolin-1 null MEFs

**Figure 5:**
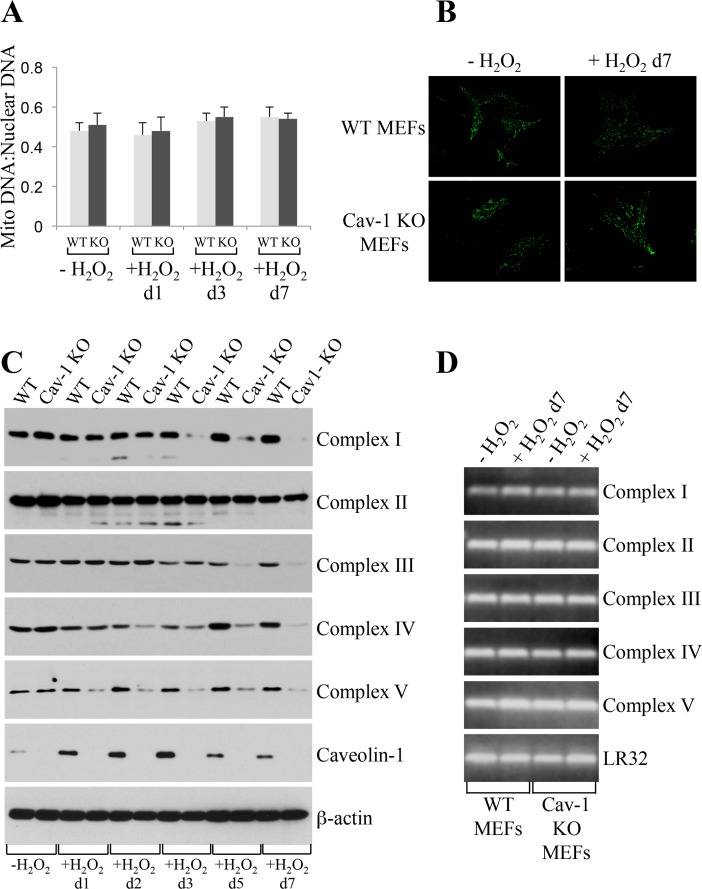
Oxidative stress promotes degradation of mitochondrial respiratory chain complexes in caveolin-1 null MEFs Wild type and caveolin-1 null mouse embryonic fibroblasts (MEFs) were treated with sublethal doses of hydrogen peroxide (150 μM) for 2 hours. Cells were then recovered in complete medium for different periods of time. Untreated cells (−H_2_O_2_) were used as control. (**A**) The ratio of mitochondrial to nuclear DNA was quantified by performing RT-PCR analysis for the mitochondrial gene ND1 and the nuclear encoded gene Histone 19 using gene-specific primers. (**B**) Cells were incubated with Mitotracker Green FM (Thermo Fisher Scientific; Waltham, MA) at a concentration of 100 nM in DMEM. Cells were incubated at 37°C for 30 min, washed with PBS and imaged using a Zeiss Confocal Microscope (LSM 5 Pascal; Carl Zeiss, Jena, Germany). (**C**) The expression level of complex I, complex II, complex III, complex IV, complex V and caveolin-1 was determined by immunoblotting analysis using specific antibody probes. Immunoblotting with anti-β-actin IgGs was performed as internal control. (**D**) RT-PCR analysis for complex I, complex II, complex III, complex IV and complex V was performed using gene-specific primers. RT-PCR analysis using primers for LR32 was performed as internal control.

**Figure 6:**
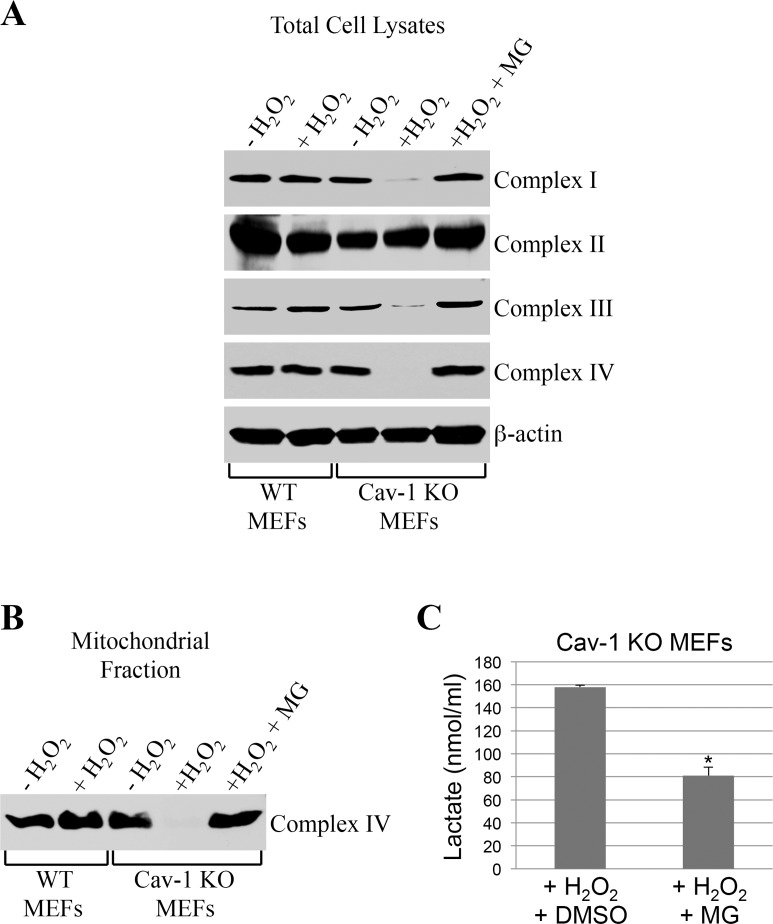
Proteasome inhibition rescues the expression of respiratory chain complexes in ROS-treated caveolin-1 null MEFs Wild type and caveolin-1 null mouse embryonic fibroblasts (MEFs) were treated with sublethal doses of hydrogen peroxide (150 μM) for 2 hours in the presence or absence of 0.1 μM MG-132. Cells were then recovered in complete medium for 7 days in the presence or absence of 0.1 μM MG-132. Untreated cells (−H_2_O_2_) were used as control. (**A**) Total expression of complex I, complex II, complex III and complex IV was determined by immunoblotting analysis using specific antibody probes. Immunoblotting with anti-β-actin IgGs was performed as internal control. (**B**) Mitochondria were isolated and the expression of complex IV was determined using anti-complex IV IgGs. (**C**) Lactate production was quantified using the Lactate Assay Kit from Sigma-Aldrich (MAK064). Values were normalized to cell number. Values in (**C**) represent mean ± SEM; *P<0.001.

How can we explain, at the molecular level, the inhibition of oxidative phosphorylation observed in caveolin-1 null MEFs? We first established that the bioenergetic defects observed in caveolin-1 null MEFs were not the consequence of either reduced number of mitochondria or altered mitochondrial morphology, as demonstrated by mitochondrial DNA:nuclear DNA ratio (Figure 5A) and Mitotracker Green FM staining (Figure 5B), respectively. Since defective mitochondrial protease activity has been linked to disrupted assembly of mitochondrial respiratory chain complexes [8–12], we then tested whether a lack of caveolin-1 leads to the degradation of respiratory chain proteins. Wild type and caveolin-1 null MEFs were left untreated or treated with subcytotoxic levels of oxidative stress and recovered for different periods of time. We demonstrate by immuno-blotting analysis that a lack of caveolin-1 did not alter the expression of complex I, II, III, IV and V under resting conditions (Figure 5C). However, with the exception of complex II, we observed a time-dependent downregulation, to almost undetectable levels, of all respiratory chain complexes after oxidative stress only in caveolin-1 null MEFs (Figure 5C). Loss of complex I, III, IV and V protein expression in caveolin-1 null MEFs was not the consequence of transcriptional defects in these cells, as demonstrated by comparable mRNA levels in wild type and caveolin-1 null MEFs before and after oxidative stress (Figure 5D). Interestingly, treatment with the proteasome inhibitor MG-132 restored the expression (Figure 6A) and the mitochondrial localization (Figure 6B) of respiratory chain proteins in oxidant-treated caveolin-1 null MEFs. Importantly, MG-132 treatment significantly reduced glycolysis in caveolin-1 null MEFs after oxidative stress (Figure 6C), suggesting that restoration of respiratory chain complexes prevented the oxidative phosphoryla-tion to glycolysis switch observed in cells lacking caveolin-1.

### A mutant form of AFG3L2 that fails to interact with caveolin-1 promotes degradation of complex IV

**Figure 7:**
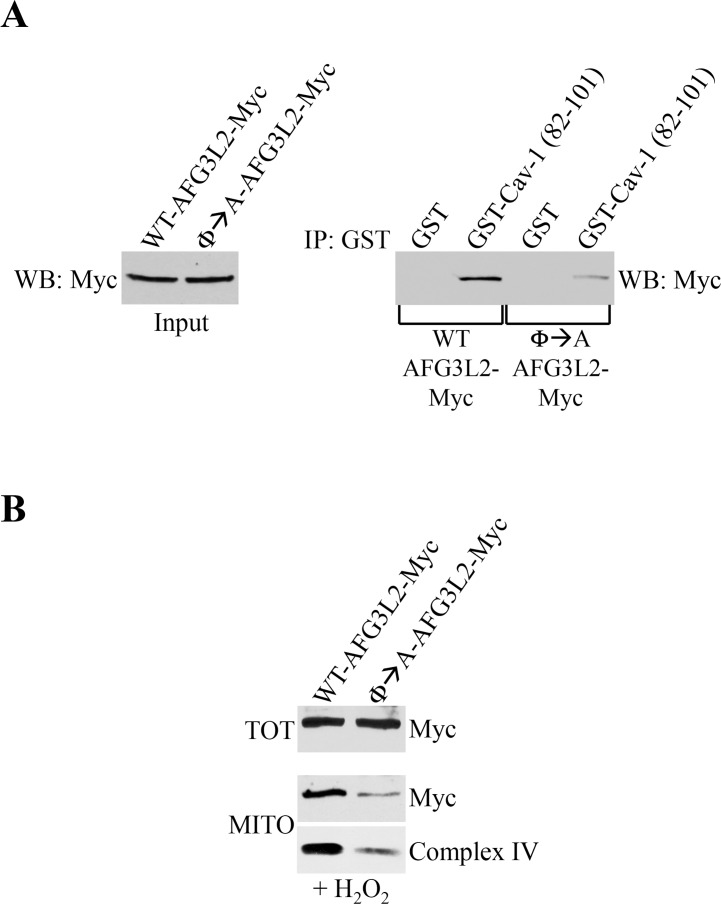
Φ→A-AFG3L2 poorly interacts with caveolin-1, does not accumulate in mitochondria and promotes degradation of complex IV after oxidative stress (**A**) GST-Cav-1(82-101) was used in pull down assays with cell lysates from NIH 3T3 cells transiently transfected with either wild type AFG3L2-myc or Φ→A-AFG3L2-myc. Pull-down assays with GST alone was used as internal control. (**B**) Wild type mouse embryonic fibroblasts (MEFs) were infected with a lentiviral vector (pLVX) expressing either WT-AFG3L2-myc or Φ→A-AFG3L2-myc. After 48 hours, cells were treated with sublethal doses of hydrogen peroxide (150 μM) for 2 hours. Cells were then recovered in complete medium for 7 days. Mitochondrial fractions (MITO) were isolated and the expression levels of AFG3L2-myc and complex IV were measured by immunoblotting analysis. Total expression (TOT) of WT-AFG3L2-myc and Φ→A-AFG3L2-myc is shown in the upper panel.

Our data show that AFG3L2 accumulates in mitochondria under conditions of sublethal oxidative stress, where AFG3L2 interacts with caveolin-1. Our findings also demonstrate that AFG3L2 fails to localize to mitochondria and respiratory chain complexes are degraded in caveolin-1-lacking cells. To prove a causal link between a lack of caveolin-1-mediated mitochondrial localization of AFG3L2 and degradation of respiratory chain complexes, we generated a mutant form of AFG3L2 (Φ→A-AFG3L2) in which the aromatic residues within the caveolin-1-binding domain were substituted with alanines (Figure 1A). We find that the interaction between Φ→A-AFG3L2 and caveolin-1 was dramatically compromised, as compared to wild type AFG3L2 (WT-AFG3L2) (Figure 7A). We then expressed either WT-AFG3L2 or Φ→A-AFG3L2 in wild type MEFs and evaluated their mitochondrial localization after oxidative stress. We show in Figure 7B that the accumulation of Φ→A-AFG3L2 in mito-chondria, in contrast to WT-AFG3L2, was significantly reduced. In addition, the reduced mitochondrial localization of Φ→A-AFG3L2 resulted in degradation of complex IV after oxidative stress (Figure 7B). These findings causally link the absence of caveolin-1-mediated targeting of AFG3L2 to mitochondria in caveolin-1 null MEFs with respiratory chain protein degradation. These data are also consistent with the notion that, in the absence of mitochondrial *m*-AAA activity, mitochondrial protein quality control of ROS-damaged proteins is prevented, leading to mitochondrial protein degradation [6, 8–12].

## DISCUSSION

**Figure 8:**
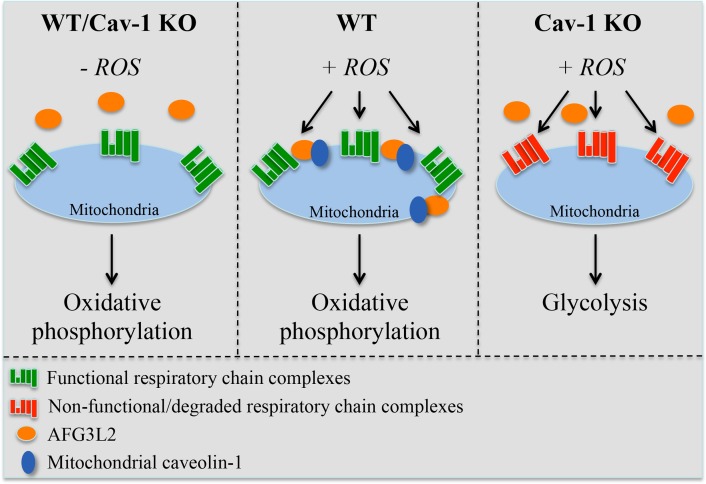
Schematic diagram summarizing the control of mitochondrial functions by caveolin-1 through the regulation of AFG3L2 Under resting conditions (-ROS), both wild type and caveolin-1 null cells possess functional respiratory chain complexes and generate energy mostly through oxidative phosphorylation. Upon oxidative stress, the caveolin-1-dependent localization of AFG3L2 to mitochondria in wild type cells prevents ROS-mediated mitochondrial damage by providing mitochondrial protein quality control. As a conse-quence, functional respiratory chain complexes are maintained. After oxidative stress but in the absence of caveolin-1, AFG3L2 fails to localize to mitochondria and the AFG3L2-mediated protective mechanism is lost, leading to the degradation of respiratory chain proteins. Under these conditions, oxidative phosphorylation is impaired and caveolin-1 null cells rely on enhanced glycolysis for their bioenergetic requirements.

Our understanding of the molecular mechanisms through which eukaryotic cells protect mitochondria against oxidative stress remains limited. Here, we describe a novel regulatory mechanism that is centered on the ability of caveolin-1 to promote the mito-chondrial localization of the *m*-AAA protease following oxidative stress. The caveolin-1-mediated mitochondrial localization of *m*-AAA prevents ROS-mediated mitochondrial damage by providing mitochondrial protein quality control. As a result, the assembly of functional respiratory chain complexes is maintained even after oxidative stress. In the absence of caveolin-1, *m*-AAA fails to localize to mitochondria and such protective mechanism is lost, leading to the degradation of respiratory chain proteins. As a consequence, oxidative phosphorylation is impaired in caveolin-1 null cells, which rely on enhanced glycolysis for their bioenergetic requirements under conditions of oxidative stress (Figure 8). The protective role of the caveolin-1-dependent mitochondrial localization of *m*-AAA is corroborated by our data showing that the expression of Φ→A-AFG3L2, a mutant form of AFG3L2 that poorly interacts with caveolin-1, fails to accumulate to mitochondria after oxidative stress and acts in a dominant negative fashion by preventing mitochondrial protein quality control and promoting the degradation of complex IV. We also found that the expression of mitochondrial respiratory chain proteins is normal in caveolin-1 null fibroblasts under resting conditions. This result suggests that, in the absence of exogenous ROS challenges, the need for *m*-AAA-dependent mitochondrial protein quality control is limited. This conclusion is supported by our data showing that AFG3L2 is only marginally localized in mitochondria before oxidant stimulation even in wild type MEFs. Thus, our findings add novel molecular insights into how eukaryotic cells respond to oxidative stress. We propose that caveolin-1 acts as a “guardian” of mitochondrial integrity when cells are attacked by exogenous ROS.

This scenario is consistent with recent findings showing that a loss of caveolin-1 expression in breast cancer cells activates nuclear erythroid 2 p45-related factor-2 (Nrf2) and promotes manganese-dependent superoxide dismutase (MnSOD)-induced glycolysis and that reconstitution of caveolin-1 expression in caveolin-1-negative breast cancer cells suppresses Nrf2, reduces MnSOD expression, inhibits glycolysis and enhances mitochondria-dependent ATP production [41]. Interestingly, we have previously demonstrated that caveolin-1 is an endogenous inhibitor of Nrf2 and that Nrf2 is hyperactivated in caveolin-1 null MEFs [28]. One could speculate that Nrf2-mediated upregulation of MnSOD might contribute to the increased glycolysis that we observe in caveolin-1 null MEFs. Moreover, our current results are supported by previous data showing that overexpression of caveolin-3, a muscle-specific caveolin isoform, in cardiac myocytes increases the mitochondrial localization of caveolin-3 and improves respiratory functions in these cells [34, 37], while a lack of caveolin-3 expression in caveolin-3 null mice leads to mitochondrial dysfunctions in the heart [34].

The proteasome is a cytosolic machinery that mediates the degradation of a variety of proteins, including those of cytosolic, endoplasmic reticulum and mitochondrial outer membrane origin. Less is known about the proteasome-mediated degradation of mitochondrial inner membrane proteins. MG-132 is a specific and potent proteasome inhibitor. Our results show that treatment with MG-132 rescues the degradation of mitochondrial respiratory chain proteins in caveolin-1 null MEFs after oxidative stress. We envision a scenario in which the loss of protein quality control in oxidant-stimulated and caveolin-1-lacking cells, due to the inability of *m*-AAA to localize to mitochondria in the absence of caveolin-1, prevents the homeostatic elimination of respiratory chain proteins that are damaged by oxidative stress. As such, the overall assembly of respiratory chain complexes is destabilized, similarly to what reported in animal models and humans lacking *m*-AAA expression. As a consequence, respiratory chain proteins are either transported into the cytoplasm to be degraded by the proteasome or made available to the proteasome machinery while still in the mitochondria. In support of this scenario, there is evidence showing that mitochondrial uncoupling protein 2 (UCP2), a mitochondrial inner membrane protein, is degraded by the cytosolic 26S proteasome.

In humans, loss of function mutations in either AFG3L2 or paraplegin lead to neuropathies associated with mitochondrial dysfunctions such as reduced complex I activity due to defective complex assembly [6, 7, 42–44]. Consistent with these observations, AFG3L2 deficient mice display defects in mitochondrial respiration and suppression of complex I assembly and activity [45–49]. In addition, mitochondrial protein synthesis and impaired mitochondrial ribosome assembly have been reported in AFG3L2 null mice [50]. Thus, genetics studies in humans and mice support our data and conclusion that altered *m*-AAA activity results in mitochondrial dysfunctions.

Mitochondrial- and nuclear-encoded respiratory chain subunits are assembled stoichiometrically in the mitochondrial membrane; as such, imbalances between subunits have the potential to disrupt mitochondrial integrity and function. A very intriguing observation is that complex II was the only mitochondrial respiratory complex that was not degraded in caveolin-1 null cells after oxidative stress. Interestingly, complex II is also the only respiratory complex that is entirely composed of nuclear DNA-encoded subunits that are translated in the cytoplasm. One possible explanation for the lack of complex II degradation in caveolin-1-lacking fibroblasts is that oxidant-induced damage of complex II subunits may occur in the cytoplasm. As such, complex II subunit quality control may be mediated by a protease that does not require the caveolin-1-mediated mitochondrial localization. An alternative explanation is that complex II is less susceptible to ROS-induced damage.

Lisanti and colleagues have shown that downregulation of caveolin-1 expression in cancer-associated fibroblasts leads to increased expression of glycolysis regulators and glycolytic enzymes therefore creating a lactate- and pyruvate-rich microenvironment [18]. These high-energy metabolites are then transferred to adjacent cancer cells and used by them to bust oxidative phosphorylation and ATP production to promote cancer cell proliferation [30, 51–53]. Based on these observations, the Lisanti's group has advanced the novel paradigm of reverse Warburg effect, based on which aerobic glycolysis does not take place in cancer cells but instead in cancer-associated stromal cells. Our studies provide novel molecular insights into the switch from oxidative phosphorylation to glycolysis, following caveolin-1 downregulation, in fibroblasts and support Lisanti's observations. We show that a lack of mitochondrial protein quality control in the absence of caveolin-1 expression leads to respiratory chain dysfunctions and enhanced glycolysis. We speculate that downregulation of caveolin-1 in cancer-associated fibroblasts may prevent the localization of *m*-AAA in mitochondria and *m*-AAA-dependent mitochondrial protein quality control with the consequent degradation of respiratory chain complexes and switch to aerobic glycolysis.

## METHODS

### Ethics statement

Investigation has been conducted in accordance with the ethical standards and according to the Declaration of Helsinki and according to national and international guidelines and has been approved by the authors' institutional review board.

### Materials

Antibodies and their sources were as follows: anti-caveolin-1 IgG (pAb N-20), anti-c-Myc (mAb 9E10) and anti-β-actin (mAb C4) were from Santa Cruz Biotechnology (Santa Cruz, CA); anti-complex I (mAb MS112), anti-complex II (mAb MS204) and anti-complex V (mAb MS502) were from MitoSciences (Eugene, OR); anti-complex IV (mAb A21348) was from Thermo Fisher Scientific (Waltham, MA); anti-complex III (mAb ab14745) was from abcam (Cambridge, MA). All other biochemicals used were of the highest purity available and were obtained from regular commercial sources.

### Cell culture and oxidative stress

Mouse embryonic fibroblasts (MEFs) were derived from wild type and caveolin-1 null mice as previously described [19]. MEFs were grown in Dulbecco's Modified Eagle's Medium (MEM) supplemented with glutamine, antibiotics (penicillin and streptomycin), and 10% fetal bovine serum. Oxidative stress was induced by subcytotoxic levels of hydrogen peroxide (150μM) for 2 hours. Cells were then recovered in normal medium for different periods of time (see text for details).

### GST fusion proteins pull-down assay

The GST-caveolin-1 (GST-Cav-1) fusion protein constructs were transformed into *Escherichia coli* (BL21 strain; Novagen, Inc.). After induction of expression through addition of 5 mM isopropyl-β-D-galactoside (Sigma), GST-Cav-1 constructs were affinity purified on glutathione-agarose beads, using the detergent Sarcosyl for initial solubilization. GST-Cav-1 and GST alone (bound to glutathione-agarose beads) were washed 3 times with TNET buffer (50 mM Tris, pH 8.0, 150 mM NaCl, 5 mM EDTA, 1% Triton X-100) containing protease inhibitors. SDS-PAGE followed by Comassie staining was used to determine the concentration of GST-Cav-1 per 100 μl of packed bead volume. Pre-cleared cell lysates were diluted in buffer A (10 mM Tris, pH 8.0, 0.1% Tween 20) and added to approximately 100 μl of equalized bead volume for overnight incubation at 4°C. After binding, the beads were extensively washed with phosphate-buffered saline (6 times). Finally, the beads were resuspended in 3X sample buffer and subjected to SDS-PAGE.

### Immunoblotting

Cells were collected in boiling sample buffer. Cellular proteins were resolved by SDS-PAGE (12.5% acrylamide) and transferred to BA83 nitrocellulose membranes (Schleicher & Schuell, Keene, NH). Blots were incubated for 2 h in TBST (10 mM Tris-HCl, pH 8.0, 150 mM NaCl, 0.2% Tween 20) containing 2% powdered skim milk and 1% bovine serum albumin (BSA). After three washes with TBST, membranes were incubated for 2 h with the primary antibody and for 1 h with horseradish peroxidase-conjugated goat anti-rabbit/mouse IgG. Bound antibodies were detected using an ECL detection kit (Pierce, Rockford, IL). Representative images of three independent experiments are shown.

### Co-Immunoprecipitation

Cells were washed twice with PBS and lysed for 30 min at 4°C in a buffer containing 10 mM Tris, pH 8.0, 0.15 M NaCl, 5 mM EDTA, 1% Triton X-100, and 60 mM octyl glucoside. Samples were precleared for 1 h at 4°C using protein A-Sepharose (20 μl; slurry, 1:1) and subjected to overnight immunoprecipitation at 4°C using the intended antibody and protein A-Sepharose (30 μl; slurry, 1:1). After three washes with the immunoprecipitation buffer, samples were separated by SDS-PAGE (12.5% acrylamide) and transferred to nitrocellulose. Then, blots were probed with the intended antibody. Experiments were performed three independent times and representative images are shown.

### RNA isolation and RT-PCR

Cells were collected and total RNA was isolated using the RNeasy Mini kit from Qiagen (Valencia, CA). Equal amounts of RNA were treated with RNase-free DNase, and subjected to reverse transcription using the Advantage RT-for-PCR kit from Clontech (Mountain View, CA), according to the manufacturer's recommendations. PCR was then performed in the exponential linear zone of amplification for each gene studied. Gene-specific primers used were as follows: complex I forward tacgacgatgaggtaaagcgg; complex I reverse tctccagcttcgagcttgaga; complex II forward ctcggaaggagtcccggggag; complex II reverse agtcagcctcattcaaggtct; complex III forward ccaagaacaagctaaaagctg; complex III reverse agtttccactcgctgccattg; complex IV forward ccagggatgagaaagttcagt; complex IV reverse gatggccacccagtcacgatc; complex V forward gaccgagttgctaaagcaagg; complex V reverse tctgactgttctgagattttc. Sequences corresponding to LR32 were also amplified as internal controls.

### Isolation of mitochondrial fraction

Cells (>5×10^7^) were washed with isolation medium (250mM Sucrose, 20 mM HEPES, 10mM KCl, 1.5mM Mg_2_Cl_2_, 1mM EDTA, 1mM EGTA pH 7.4) at 4°C and scraped in 5 volumes of isolation medium. Cells were homogenized using a glass on glass dounce homogenizer (~20 strokes). Cell suspension was then centrifuged at 750 x g for 10 min. The supernatant was collect and centrifuged at 10,000 x g for 15 min. The resulting pellet was resuspended in isolation medium and comprised active mitochondria.

### Mitochondria imaging

Live cells were washed with PBS. Cells were then incubated with either Mitotracker Green FM or Mitotraker Red CMXRos (Thermo Fisher Scientific; Waltham, MA) at a concentration of 100 nM in DMEM. Cells were incubated at 37°C for 30 min, washed with PBS and imaged using a Zeiss Confocal Microscope (LSM 5 Pascal; Carl Zeiss, Jena, Germany). Quantification of signal intensity was performed with Image J software.

### Measurement of mitochondrial protease activity

Mitochondria were isolated as described above. Mitochondrial protease activity was quantified using the Protease Fluorescent Detection Kit from Sigma-Aldrich (St. Louis, MO) (PF0100), according to the manufacturer's recommendations.

### Measurement of lactate production

Lactate production was quantified using the Lactate Assay Kit from Sigma-Aldrich (MAK064), according to the manufacturer's recommendations.

### Measurement of ATP production

ATP production was quantified using the Adenosine 5′-triphosphate (ATP) Bioluminescent Assay Kit from Sigma-Aldrich (FL-AA), according to the manufac-turer's recommendations.

### DAPI (4,6-Diamidino-2-Phenylindole) staining

MEFs were harvested and incubated with staining buffer (PBS containing 3.7% paraformaldehyde, 0.1% Triton, 10 μg/ml RNase A and 1 μg/ml DAPI) at room temperature for 1 hr. Nuclear morphology was examined under a Olympus Provis fluorescent microscope. A total of 500 cells were scored from 10 independent viewing areas from three independent experiments for each experimental point.

### Oxygen consumption rate

Oxygen consumption rate was measured by Seahorse XF analysis as previously described [54]. Briefly, MEFs (32,000 cells/well) were treated in XF24 microplates. Media was changed to un-buffered DMEM after treatment and the plate incubated in a non-CO_2_ incubator (37°C; 60 min) before running on the XF24 Analyzer (Seahorse Bioscience). Basal OCR was measured after pre-warmed pharmacological modulators oligomycin (1μM), FCCP (2μM), 2-DG (100mM) and antimycin A (5μM) were injected into each well. Viability was assessed by crystal violet staining and OCR normalized to cell number.

### Mitochondrial ROS measurement

Mitochondrial ROS production was measured by MitoSOX fluorescence in intact MEFs. Cells were loaded with MitoSOX red (5μM) and fluorescence (excitation 510nm/emission 580nm) was read for 30 minutes in the presence and absence of the uncoupler FCCP (5μM). The rate of production was normalized to cell number.

### Mitochondrial DNA quantification

The ratio of mitochondrial to nuclear DNA was quantified by first isolating total cellular DNA and then performing RT-PCR for a mitochondrial gene (ND1) and a nuclear encoded gene (Histone 19). The ratio of the genes was calculated. The primers were as follows: MT-ND1 forward aat cgc cat agc ctt cct aac at; MT-ND1 reverse ggc gtc tgc aaa tgg ttg taa; H19 forward gta ccc acc tgt cgt cc; H19 reverse gtc cac gag acc aat gac tg.
